# Inflammatory and Angiogenic Protein Detection in the Human Vitreous: Cytometric Bead Assay

**DOI:** 10.1155/2011/459251

**Published:** 2011-12-27

**Authors:** M. J. Koss, M. Pfister, F. H. Koch

**Affiliations:** Section of Vitreo-Retinal Surgery, Department of Ophthalmology, Hospital of the Goethe University, Goethe University, 60590 Frankfurt am Main, Germany

## Abstract

*Introduction.* To evaluate clinical feasibility and reproducibility of cytometric bead assay (CBA) in nondiluted vitreous samples of patients with age-related macular degeneration (ARMD), diabetic macular edema (DME), and central retinal vein occlusion (CRVO). *Methods.* Twelve patients from a single clinics day qualified for intravitreal injections (ARMD *n* = 6, DME *n* = 3, CRVO *n* = 3) and underwent a combination treatment including a single-site 23 gauge core vitrectomy which yielded a volume of 0.6 mL undiluted vitreous per patient. Interleukin-6 (IL-6), vascular endothelial growth factor isoform A (VEGF-A), and monocyte chemo-attractant protein-1 (MCP-1) were assessed directly from 0.3 mL at the same day (fresh samples). To assess the reproducibility 0.3 ml were frozen for 60 days at −80°, on which the CBA was repeated (frozen samples). *Results.* In the fresh samples IL-6 was highest in CRVO (median IL-6 55.8 pg/mL) > DME (50.6) > ARMD (3.1). Highest VEGF was measured in CRVO (447.4) > DME (3.9) > ARMD (2.0). MCP-1 was highest in CRVO (595.7) > AMD (530.8) > DME (178). The CBA reproducibility after frozen storage was examined to be most accurate for MCP1 (*P* = 0.91) > VEGF (*P* = 0.68) > IL-6 (*P* = 0.49). *Conclusions.* CBA is an innovative, fast determining, and reliable technology to analyze proteins in fluids, like the undiluted vitreous, which is important to better understand ocular pathophysiology and pharmacology. There is no influence of intermittent storage at −80° for the reproducibility of the CBA.

## 1. Introduction

The measurement of ocular inflammatory and angiogenic cytokines has become increasingly important in the era of intravitreal drug therapy in many retinal diseases, primarily age-related macular degeneration (ARMD), diabetic macular edema (DME), and retinal vein occlusion (RVO) [1–3]. In the field of ophthalmology, clinical and preclinical studies are up to date based primarily on ELISA (enzyme-linked immunosorbent assay) results, which have been utilized extensively to understand the expression of cytokines from the ocular tissues (choroid, retinal pigment epithelium, retina). The quantitative analysis of a single factor analysis, however, requires for each measurement about 0.4 cc of fluid and 360 minutes of laboratory time. A new, innovative technique, the cytometric bead array (CBA) technology, provides in contrast a quantitative analysis of multiple markers in various specimen, requires a smaller sample volume, and is time- and cost-effective [4]. It is based on the flow cytometry, which is an analytical tool that allows a discrimination of different particles on the basis of size and/or color.

It has been shown earlier, using serum or blood samples, that CBA and ELISA results correlated well [5]. It is matter of debate, however, if CBA and ELISA values of undiluted vitreous are comparable under various clinical and laboratory conditions in ophthalmology. Until today, Maier et al. were the only ones to demonstrate that vitreous and serum concentrations of cytokines, including vascular endothelial growth factor (VEGF), determined by CBA showed a strong correlation with those measured by ELISA in patients with advanced diabetic retinopathy that had to undergo three-port vitrectomy [6]. Additionally it is not clear, from a laboratory point of view, if the CBA results are reproducible after intermittent storage at −80°C, which often happens in the clinical and laboratory routine. Furthermore it is interesting to assess the CBA technique for the detection of inflammatory and proangiogenic cytokines in patients that would qualify for an intravitreal injection with anti-VEGF, steroids, or a combination therapy of both agents due to a vision compromising macular edema. The majority of data derives from aqueous samples so far [7], which is important as aqueous samples should correlate well with the vitreous samples, but the absolute concentrations of cytokines in the two fluids differ significantly [8]. Before establishing a new method like the CBA in ophthalmology, it is important to evaluate the clinical feasibility and reproducibility with vitreous fluid.

In this study concentrations of interleukin-6 (IL-6), vascular endothelial growth factor (VEGF), and monocyte chemoattractant protein-1 (MCP-1) were analyzed from undiluted, cut vitreous samples from patients with age related macular degeneration (ARMD), diabetic macular edema (DME), and central retinal vein occlusion (CRVO), which qualified for intravitreal injections on one clinical day.

## 2. Methods

### 2.1. Study Population

Twelve patients were included in the present study: six age-related macular degeneration (ARMD) patients (age 77.9 ± 6.9 years) with an active occult choroidal neovascularization and an average central macular thickness of 368 ± 45 *μ*m measured by spectral optical coherence tomography (OCT; 3D OCT-2000; Topcon Deutschland, Willich, Germany), three patients with diabetic macular edema (DME; age 66.8 ± 10.5 years) and a mean central macular thickness of 508 ± 108 *μ*m, and three patients (69.5 ± 14.2) with central retinal vein occlusion (CRVO) with a history of 11 weeks ± 3 weeks of CRVO and a central macular thickness of 557 ± 125 *μ*m. The indications for the treatment were visual acuity decreasing macular edema adhering to our concept of combined therapy [9, 10].

Exclusion criteria for this series of patients were previous intraocular surgery, acute ocular infection, uveitis, trauma, vitreous hemorrhage, and retinal detachment.

Collection of samples was performed after local full ethics committee approval (57/08) in accordance with the European Guidelines for Good Clinical Practice and the Declaration of Helsinki. Informed consent was obtained from each patient before the start of therapy.

### 2.2. Statistical Analysis

Data were analyzed using Bias software (Version 8.3.8, Epsilon, Darmstadt, Germany) for Windows. All tests were performed at an error level of 5%. David's test was used to check for distribution of data. To determine the influence of intermittent storage the nonparametric Wilcoxon-matched pair-test was used including the Hodge-Lehmann indicator, which compares the variance of the results tested with the 95% confidence interval.

### 2.3. Sample Collection and Preparation

A limited core pars plana vitrectomy was performed using a single-site 23 gauge vitrector (Intrector, Insight Instruments, Stuart, FL, USA), which has two separate channels for aspiration and infusion. After conjunctival displacement an oblique sclerotomy was performed to illuminate the tip of the vitrector with a head-set and a magnifying 28 diopters lens in the mid-vitreous cavity. An assistant then aspirated a total of 0.6 mL undiluted and cut mid and posterior vitreous instructed by the surgeon, who controlled clinical relevant perioperative hypotonia. Thus a minimum sample volume of 0.3 mL vitreous was available for “fresh” direct CBA analysis on the same day and 0.3 mL “frozen” samples were available for storage at −80°C to test the CBA reproducibility. At the end of the limited posterior core vitrectomy a subsequent isovolumic substitution of balanced salt solution (BSS, ALCON, Freiburg, Germany, 0.3 mL), 1.25 mg (0.1 mL) bevacizumab (Avastin, Genentech, San Fransisco, CA, USA), and 0.8 mg (0.2 mL) dexamethasone (Dexa-ratiopharm, Ulm, Germany) was injected adhering to the concept of combination treatment in these retinal diseases [9, 10].

### 2.4. Cytokines

#### 2.4.1. IL-6

Interleukin-6 (IL-6) is a mediating protein during inflammation processes and the maturation of B cells. The protein is primarily produced at sites of acute and chronic inflammation, where it is secreted into the serum and induces a transcriptional inflammatory response through IL-6 receptor-alpha, which is associated with VEGF expression [11]. In ophthalmology it is described in a wide variety of inflammation-associated disease states what clinically can be seen as a macular edema during DME and RVO [1, 3, 12].

#### 2.4.2. VEGF

Vascular endothelial growth factor (VEGF) under physiologic normal conditions is expressed in the RPE for the retinal metabolism and balances the fenestration and the nutrition of the choriocapillaris [13]. Overexpression of VEGF, however, is induced in retinal cells mainly by hypoxia and IL-6 and leads to increased vascular permeability [11], like in a choroidal neovascularization, diabetic retinopathy, and furthermost in retinal vein occlusion [14]. It may seem that the expression of VEGF is associated with the total area of ischemic retina [15].

#### 2.4.3. MCP-1

Monocytic chemotactic protein-1 (MCP-1) is a member of the CC chemokines (Cystein-cystein chemokines). Immune cells, such as monocytes, are recruited by the expression of MCP-1. It is produced by retinal endothelial cells and has been implicated in leukostasis in the hypoxic retina and can thus be induced by VEGF [16, 17]. Previous data presumed MCP-1 as a potential factor in the proliferative phase of DR [18].

### 2.5. Cytometric BEAD Assay (CBA)

Amounts of VEGF-A, IL-6, and MCP-1 were determined using the cytometric bead array system (BD, Heidelberg, Germany). Experiments were carried out following the manufacture's instruction manual. In brief, 50 *μ*L of each vitreous body sample was incubated for 1 h with appropriate amounts of detection beads, which were specific for each investigated factor. Afterwards, samples were incubated for 2 h with detection reagent, which again was specific for each used detection bead. Samples were measured on a FACS Canto II (BD, Heidelberg, Germany) and analysed by FCAP array software (BD, Heidelberg, Germany). The amount of VEGF, IL-6, and MCP-1 was calculated using a specific standard curve (in pg/mL). The CBA was repeated in theses samples, that were acquired during surgery and stored at −80°C for 60 days.

## 3. Results

### 3.1. CBA

In the fresh samples IL-6 was highest in CRVO (median IL-6 55.8 pg/mL; 95% confidence interval 12.2–149.1) > DME (50.6; 3.5–89) > ARMD (3.1; 2.4–60.2). Highest VEGF was measured in CRVO (447.4; 51.8–454) > DME (3.9; 3.6–46.5) > AMD (2.0; 1.5–4.1). MCP-1 was higher in CRVO (595.7; 463.1–2397.8) > AMD (530.8; 38.3–768.3) > DME (178; 147.5–339.8; Table 1).

In the frozen samples IL-6 was highest in DME (55.7; 4.4–99.1) > CRVO (51.7; 9.9–237.7) > ARMD (4.8; 3.2–114.3). VEGF was highest in the CRVO group (414.7; 38.6–421.1) > DME (4.6; 4.4–54.5) > ARMD (2.2; 1.3–3.9). MCP-1 was highest in CRVO (601.2; 482–2055) > ARMD (306.7; 42.1–1115.3) > DME (164.4; 164.4–363.7).

None of the data were parametrically distributed (*P* = 0.67 David's test).

### 3.2. CBA Reproducibility

The values of the fresh versus frozen values were not statistically significant and thus comparable for all three proteins tested and most accurate for MCP1 (*P* = 0.91) > VEGF (*P* = 0.68) > IL-6 (*P* = 0.49). Additionally, the difference of Hodge-Lehmann, which indicates the overall median deviation of the specific protein levels, proves that there is no significant difference in factor levels after storage (Table 2). In general, there is no degeneration of vitreous IL-6, MCP-1, and VEGF caused by storage at −80°C for 60 days.

## 4. Discussion

This study was intended to evaluate the feasibility and reproducibility of cytometric bead assay (CBA) in a routine clinics and laboratory day setting. It was not intended to measure cytokine levels per disease, as the major limitation of this study is the small sample size. We demonstrated feasibility to assess the undiluted vitreous samples of 12 patients by performing surgery in the morning, aspirate 0.6 mL per patient, and analyze 0.3 mL of the probes directly on the same day three hours later. We observed cytokine levels for RVO and DME that were in accordance with the literature. There is to the best of our knowledge so far no evidence on the levels of intraocular cytokines of undiluted vitreous samples in AMD. In theory a greater number of fresh AMD samples could easily be gained on the basis of our protocol from a day's surgical program. In a clinical routine, however, it is questionable if enough fresh samples can be acquired and therefore intermittent storage needs to be evaluated. The other 0.3 mL of the probes were therefore frozen at −80 intermittent storage to later test if the results are reproducible. We could not detect any statistical significant degeneration of cytokines (IL-6 *P* = 0.49; VEGF *P* = 0.68; MCP-1 *P* = 0.91) and conclude that storage of vitreous samples does not alter the cytokine levels and that results of previously stored and immediately analyzed samples are comparable.

There is a large body of evidence indicating that cytokines mediating angiogenic and inflammatory processes in the choroid, the RPE, and the retina, like IL-6, VEGF, and MCP-1, contribute to the pathogenesis of ARMD, RVO, and DME [7, 19, 20]. Additionally it could be demonstrated that, in vitreous samples of patients that qualified for three-port pars plana vitrectomy, due to proliferative disease conversion, the concentration of these angiogenic factors is increased, which was demonstrated by the majority of cases with ELISA [1, 3]. We are aware, however, that this small series of patient samples without a control group and that it is not able draw conclusions about cytokines in these diseases. Instead we could demonstrate that cytometric Bead Assay (CBA) is a time- and cost-effective tool and—in comparison to ELISA—a powerful technique to analyze a larger number of samples in various retinal diseases. In comparison to ELISA only one cytokine could have been analyzed from the sample size in our study, would have generated more time, and would thus have been more expensive.

## 5. Conclusion

In AMD, DME, and RVO the clinical importance of frequent intravitreal anti-VEGF or steroid therapy, which is drawn upon clinical observation with OCT or fluorescein angiography and not upon a direct biological feedback mechanism, warrants the evaluation of new analytical techniques like CBA.

##  Conflict of Interests

None of the authors have a conflict in the subject presented.

## Figures and Tables

**Table 1:** Protein values in pg/mL of AMD (*n* = 6), DME (*n* = 3), and CRVO (*n* = 3) patients.

*N* = 12	Fresh samples	Frozen samples
	*Median 1.Quartile/3.Quartile*	*Median 1.Quartile/3.Quartile*

IL-6	10.8	10.4
	2.5/249.1	3.2/237.7

ARMD (*n* = 6)	3.1	4.8
	2.4/60.2	3.2/114.3
DME (*n* = 3)	50.6	55.7
	3.5/89.0	4.4/99.1
CRVO (*n* = 3)	55.8	51.7
	12.2/149.1	9.9/237.7

VEGF	4.0	4.1
	1.5/454.0	1.3/421.1

ARMD (*n* = 6)	2.0	2.2
	1.5/4.1	1.3/3.9
DME (*n* = 3)	3.9	4.6
	3.6/46.5	4.4/54.5
CRVO (*n* = 3)	447.4	414.7
	51.8/454.0	38.6/421.1

MCP-1	435	395.9
	42.9/2397.8	42.1/2055.5

ARMD (*n* = 6)	530.8	306.7
	38.3/768.3	42.1/1115.3
DME (*n* = 3)	178.0	164.4
	147.5/339.8	164.4/363.7
CRVO (*n* = 3)	595.7	601.2
	463.1/2397.8	482.0/2055.5

**Table 2:** Comparison of fresh samples and frozen stored samples using the Wilcoxon-matched pair-test including the Hodges-Lehmann indicator.

	IL-6 fresh versus frozen	VEGF fresh versus frozen	MCP-1 fresh versus frozen
	*P* = 0.49	*P* = 0.68	*P* = 0.91

Difference of Hodges-Lehmann	−0.85	−1.9	2.5
